# Perceived extrinsic barriers hinder community detection and management of mild cognitive impairment: a cross-sectional study of general practitioners in Shanghai, China

**DOI:** 10.1186/s12877-022-03175-4

**Published:** 2022-06-09

**Authors:** Yuan Lu, Chaojie Liu, Sally Fawkes, Zhaoxin Wang, Dehua Yu

**Affiliations:** 1 Department of General Practice, Yangpu Hospital, Tongji University School of Medicine, Shanghai, 200090 China; 2 School of Psychology and Public Health, La Trobe University, Melbourne, VIC 3086 Australia; 3 Tongji University School of Medicine, Shanghai, 200092 China; 4 Shanghai General Practice and Community Health Development Research Center, Shanghai, 200090 China; 5 School of Public Health, Shanghai Jiaotong University School of Medicine, Shanghai, 200025 China

**Keywords:** Mild cognitive impairment, Structural equation model, General practitioner, Primary care, Extrinsic barriers

## Abstract

**Background:**

General practitioners (GPs) play a critical role in community detection and management of mild cognitive impairment (MCI). Although adequate knowledge is essential, healthcare practice is shaped by intrinsic and extrinsic factors. This study aimed to test the mediating effect of perceived extrinsic barriers on the associations between knowledge, attitudes, and intended practice of GPs in community detection and management of MCI.

**Methods:**

A cross-sectional study was conducted through an online survey of 1253 GPs sampled from 56 community health centres (CHCs) in Shanghai in 2021. Perceived extrinsic barriers were rated on a five-point Likert scale for patient engagement, working environment, and system context, respectively. A summed score was generated subsequently for each domain ranging from 0 to 100, with a higher score indicating higher barriers. The mediating effect of perceived extrinsic barriers (second-order) and the moderation effect of training on the association between MCI knowledge and practice scores, as well as the moderation effect of past experience on the association between MCI knowledge and extrinsic barriers, were tested through structural equation modelling (SEM) with a partial least square (PLS) approach.

**Results:**

The study participants reported an average barrier score of 65.23 (SD = 13.98), 58.34 (SD = 16.95), and 60.37 (SD = 16.99) for patient engagement, working environment, and system context, respectively. Although knowledge had both direct and indirect (through attitudes) effects on intended practice, perceived extrinsic barriers negatively mediated (β = − 0.012, *p* = 0.025) the association between knowledge and practice. Training moderated the effect of knowledge on practice (β = − 0.066, *p* = 0.014).

**Conclusions:**

Perceived extrinsic barriers have a detrimental effect on the translation of knowledge into practice for community detection and management of MCI. The effect of training on practice declines when knowledge scores become higher.

**Supplementary Information:**

The online version contains supplementary material available at 10.1186/s12877-022-03175-4.

## Background

Mild cognitive impairment (MCI) as an intermediate phase between normal cognitive ageing and overt dementia has attracted a great deal of interest in research that aims to reduce the growing burden of dementia [1]. In China, the prevalence of MCI in those aged 55 years or older has reached 17% [2]. MCI was estimated to convert to dementia at a rate of up to 20% every year if not properly managed [3]. According to the studies conducted in Europe and North America, the disease management costs would be more than doubled once the cognitive impairment condition progressed into dementia [4].

General practitioners (GPs) play a critical role in community detection and management of MCI. Early community detection and management of MCI may increase the likelihood of slowing down the fast progression of further cognitive impairment [5]. The American Academy of Neurology recommends screening of MCI in primary care settings so that most of the insidious onset of MCI in its preclinical asymptomatic phase can be detected [6]. The current evidence available regarding effective MCI management shows that GPs are placed in a unique position to support patients to manage MCI [7] because non-pharmaceutical measures such as adjustment of the modifiable risk factors [8] (e.g., smoking, diabetes, cerebrovascular disease) and cognitive interventions remain the most cost-effective strategies in MCI management [9], and all of these measures can be implemented in primary care settings. However, the cognitive problems of a significant number of patients have not been recognised by GPs in their daily practices worldwide [10], even though the majority of GPs acknowledged the value of cognitive impairment assessment in primary care [11]. The Ageing, Demographics, and Memory Study (ADAMS) in the United States (US) showed that in 845 community-based seniors over 70 years, only 8% had received a memory assessment, compared with 94% of elderly individuals reporting the benefits of early screening and intervention on dementia [12].

A wide range of factors influences the clinical practice of healthcare professionals. A knowledgeable healthcare workforce is a key to meeting the changing demands of healthcare services. However, empirical evidence shows that clinical practices of health professionals are not always aligned with their acquired knowledge [13]. They can be influenced by the individual motivational predispositions to change, as well as by the organisational, economic, social, and political contexts [13]. Previous studies show that adherence of medical doctors to practice guidelines is determined by the demands of the individual patient, the beliefs of the medical doctor, the peer culture, the management and organisational climate, health system arrangements, and the broad social environment [14, 15]. Researchers have attempted to categorise the above-mentioned determinants of practice decisions into various theoretical frameworks. The United Nations Children’s Fund (UNICEF, 2019) [16] summarised 25 behavioural theories and models. Although most of the theories have been developed to understand health behaviours of the general public, some have been adopted in studies on the practice behaviours of health professionals. For example, the US Centers for Disease Control and Prevention (CDC) Campaign to Prevent Antimicrobial Resistance Team assessed the motivation of hospital physicians to take action to prevent antimicrobial resistance in their patients in line with the health belief model (in terms of perceived susceptibility, severity, benefits, barriers, and self-efficacy) [17]. The theory of reasoned action (TRA) was also used in explaining physicians’ behaviours based on their individual attitudes (intrinsic motivation), subjective norms (perceived social pressure), and intention to act [18]. The theory of planned behaviour (TPB) is another commonly used theory to describe the intention of health professionals to use clinical guidelines, which extends the TRA by adding perceived control over behaviour as a new construct [19]. These commonly used behavioural theories examine human behaviours from different angles [20]. Nevertheless, they all acknowledge the existence of intrinsic and extrinsic drivers despite some bias towards one or the other, which aligns well with the social cognitive theories [13] that emphasise the reciprocal determination in the interaction between people and their environments.

Both intrinsic and extrinsic drivers have been deemed important to incentivise GPs to adopt and adhere to practice guidelines in MCI detection and management [7, 20]. There exist variations in the individual (intrinsic) attitudes of GPs toward community detection and management of MCI [11, 21]. Attitudinal barriers are particularly detrimental to preventive interventions such as the screening of MCI [22]. Apart from provider-related intrinsic barriers such as a lack of knowledge and confidence, a recent systematic review of 16 studies identified patient-related barriers and system-related barriers that can jeopardise the efforts of primary care physicians to provide optimal dementia care [23]. For example, patients may be reluctant to acknowledge cognitive decline and not willing to adhere to management plans; the health system may not dedicate enough resources and managers may not actually render adequate support. There is a stigma attached to dementia and cognitive impairment in society. Ageism and financial constraints are often blamed for jeopardising the rapid appraisal and management of cognitive disorders in primary care according to another systematic review of 11 studies [24]. However, there is a dearth of literature comprehensively assessing the effects of both intrinsic and extrinsic barriers on community detection and management of MCI, in particular in low- and middle-income countries.

This study aimed to address the gap in the literature by testing the mediation role of perceived extrinsic barriers on the associations between knowledge, and intended practice of GPs in community detection and management of MCI. The findings of the study will also provide evidence in support of the novel development of community-based intervention programs for MCI in China.

### Study hypotheses

**Fig. 1:**
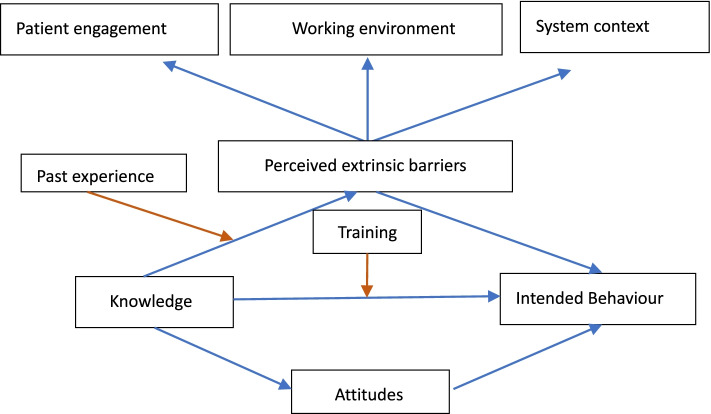
Structural model of intended practice of general practitioners in MCI detection and management

The Knowledge-Attitudes-Practices (KAP) model is arguably the most commonly used theoretical framework in examining the behaviours of health professionals. However, it has been criticised for the lack of consideration of extrinsic factors [25]. Empirical evidence shows that human behaviours are not always aligned with individuals’ knowledge [13]. The choice of actions of health professionals is also shaped by regulations, policies, rules, and pressures from consumers [26]. The importance of the influence of the organisational environment, in which a health practitioner works, has been increasingly recognised [27]. In this study, we tested the effects of several factors on the K-A-P pathway (Fig. 1).

### Hypothesis one: perceived extrinsic barriers mediate the effect of knowledge on practice

We categorised extrinsic barriers into three domains in line with the Chronic Care Model (CCM) [28]: patient engagement, working environment, and system context. The CCM aims to foster improvements in the care for patients with chronic illnesses by emphasising the importance of prepared, proactive practice teams, well-informed and motivated patients, as well as a system platform that enables effective interactions between the two. High-quality chronic illness care is characterised by a productive interaction between the practice team and its patients [29]. Patients need correct and relevant information and confidence to engage in their care, while the practice team requires time and resources to act. A supportive system fosters an appropriate climate to empower its employees to perform well. Barriers arising from these extrinsic factors can jeopardise the practice efforts of GPs in community detection and management of MCI.

### Hypothesis two: MCI training moderates the effect of knowledge on practice

Training has been considered one of the most important measures to initiate a new medical intervention program. A cohort study in the US over a two-year period found that training support was effective in improving the confidence of primary care workers in dementia care and their competency in using the cognitive screening tools [30]. Continuing education was also found to be beneficial for improving MCI detection in primary care in a study in Hungary [31]. We did not test the moderation effect of training on the association between MCI knowledge and attitudes because of the lack of significant association (*p* = 0.938) between MCI training and attitude scores.

### Hypothesis three: past experience moderates the association between knowledge and perceived extrinsic barriers

Past experience influences the level of felt easiness of clinicians in making clinical decisions [18]. It is also associated with knowledge acquisition. A cross-sectional study of 197 family physicians in Israel found that those who made MCI diagnoses over the previous 6 months reported higher levels of MCI knowledge than those who had not [32]. A knowledgeable health practitioner is more likely to be able to identify the existing extrinsic barriers [33]. Therefore, it is reasonable to assume that past experience may have a direct effect on perceived extrinsic barriers, and, in turn, may moderate the association between knowledge and perceived extrinsic barriers.

## Methods

### Study settings

This study was conducted in Shanghai, China. Shanghai ranks fourth in population ageing in China, with over 16.28% of its residents exceeding the age of 65 years [34]. As one of the earliest cities in China to transition to an ageing society, Shanghai is the first in line to develop “Friendly Community Programs” for the elderly with cognitive impairment [35] as part of the Healthy China Strategy [36]. Healthcare organisations are encouraged to work in partnerships with local community organisations in responding to the challenge of ageing, in particular in relation to cognitive impairment.

GPs have been assigned a critical role in the “Friendly Community Programs”. They are supposed to perform MCI screening and risk assessment, initiate MCI diagnosis, conduct community interventions, coordinate with other care providers, and educate the public [37]. Over the past few decades, China has attempted to revitalise its primary care system through the development of community health services. GPs, as a new medical specialist stream, serve as a backbone in China’s community health services [38]. In Shanghai, 246 community health centres (CHCs) have been established to meet the essential healthcare needs of all of its residents. About 10,000 GPs (4.12 Per capita) were employed by these CHCs in 2019 [34]. To improve the training of qualified GPs, the National Health Commission promulgated a plan to standardise medical training under a new “5 + 3” framework in 2012 [39], with 5 years of undergraduate study followed by 3 years of standardised residency training. However, training programs targeting the screening and interventions of cognitive disorders are limited.

CHCs receive funding from the local health department that supports infrastructure and population-based services (essential public health services) [40]. Individual-based medical care is usually covered by social health insurance schemes. However, patients are also charged a fee for their individual medical care, albeit at a lower rate compared with their hospital counterparts [41]. Currently, the MCI detection and management services are partly funded through the essential public health services and partly through patients and their health insurance programs. Unfortunately, at this stage, there is no additional funding coming through the “Friendly Community Programs” to support the new MCI management initiative in CHCs.

### Survey instruments

A questionnaire was developed through a thorough examination of the existing tools in relation to the testing constructs [31, 42, 43]. This was followed by focus groups interviews with 32 MCI patients and their caregivers, 42 GPs, and 18 CHC managers to adapt the tools to the specific context of China. Two rounds of Delphi consultations with 24 experts were conducted to achieve consensus on the measurements. This process ensured that the tools could capture a wide range of issues of concerns.

The questionnaire contained three sections. Section one measured the characteristics of the study participants, including their training and working experience in relation to MCI.

Section two measured the MCI-related KAP of the study participants. The KAP measurements adopted a formative structure, covering all essential elements required in community detection and management of MCI, including perceived intrinsic barriers embedded in the measurement of attitudes (in terms of perceived seriousness of the problem and benefits and self-efficacy in MCI management). A summed score was calculated for knowledge, attitudes, and practice, respectively, and transformed into a standardised score ranging from 0 to 100, with a higher score indicating a trend in favour of community detection and management of MCI. The KAP scales were validated using the four criteria recommended by Diamantopoulos and Winklhofer [44] including content specification, indicator specification, indicator collinearity, and external validity, which were reported in our previous study [45].

Section three measured perceived extrinsic barriers reflected in three domains: patient engagement, working environment, and system context. The measurement of perceived barriers in patient engagement followed the conceptual framework developed by Davis et al. through a comprehensive literature review [46]. It contained 14 items, including person-related (8 items), illness-related (2 items), healthcare professional-related (2 items), healthcare setting-related (1 item), and task-related (1 item) barriers. Perceived barriers in working environment were measured in line with the “building blocks” suggested by the World Health Organization (WHO) [47], including leadership/governance (2 items), financing (1 item), workforce (4 items), information systems (1 item), and service delivery (4 items). Perceived barriers in system context reflect the broad system and societal environments in which a health care organisation operates. The measurement was informed by the framework of structural domains for primary care developed by the Lamont Primary Health Care Research Centre in Canada [48]. It contained 7 items, including 2 items reflecting the financial and policy support from the government and the health system, respectively, and 3 items measuring acceptance of MCI detection and management from the broad society and the public media.

Study participants were asked to rate each item on a five-point Likert scale, ranging from 1 (strongly disagree) to 5 (strongly agree). A summed score was calculated for each domain and then transformed into a standardised score ranging from 0 to 100. A higher score indicates a higher level of perceived extrinsic barriers.

### Study participants and data collection

Data were collected from 56 CHCs out of 246 CHCs across all 16 districts in Shanghai during the period from 13 April to 9 May in 2021. A stratified cluster sampling strategy was applied to recruit participants in proportion to the district distribution of the CHCs. Eligible participants were registered GPs in the CHCs who had direct contact with patients.

Permission from the senior managers of the targeted CHCs was sought through emails before a survey invitation was dispatched to all of their eligible GPs. Study respondents were invited to provide implied informed consent before proceeding with the survey. The survey was anonymous and respondents could withdraw at any time.

The survey took around 15 minutes to complete via the online platform RedCAP [49]. In total, 1789 of the invited participants accessed the survey platform, with 1740 being recorded with a submission. Of the returned questionnaires, 1253 contained no missing items and were included in data analyses. This represented an effective response rate of 70.04%. The sample size was large enough for PLS-SEM modelling, which is known for its advantage of handling large numbers of items with a relatively small sample size [50].

Ethical clearance to conduct the study was obtained from the Research and Ethics Committee of La Trobe University in Melbourne, Australia (HEC20143) and Yangpu Hospital in Shanghai, China (LL-2019-SCI-004).

### Statistical analysis

Perceived extrinsic barriers were described through frequency distribution of items and using summed scores (Mean ± Standard Deviation) of the three domains: patient engagement, working environment, and system context. Pearson correlation analyses were performed to test the relationships between the MCI-related KAP scores and the three domains of perceived extrinsic barriers.

Structural equation modeling (SEM) was established to determine the mediation effect of perceived extrinsic barriers and the moderation effect of training on the association between knowledge and intended practice of GPs in community detection and management of MCI, and the moderation effect of past experience on the association between MCI knowledge and extrinsic barriers. In the SEM, the three domains of extrinsic barriers formed a second-order construct. The intended practice was also deemed a second-order construct, comprising three domains: alerting, confirming, and managing.

A partial least squares (PLS) approach was selected in the SEM because of the complex exploratory nature of the model and the inclusion of both reflective and formative constructs. PLS-SEM adopts a nonparametric method, which does not have restrictive requirements on the distribution of data [50]. In this study, two successive model assessments were performed: measurement tests followed by structural tests. The reliability of the scales measuring the three domains of extrinsic barriers was assessed using Cronbach’s alpha (> 0.7), composite reliability (CR > 0.7), and ρA (> 0.7). Items with a loading lower than 0.7 were retained if removal of the item would not increase composite reliability [50]. The convergent validity of the scales was assessed using the average variance extracted (AVE ≥ 0.50) [50]. The Heterotrait-monotrait criterion (HTMT< 0.90) was used to establish discriminant validity [51]. The structural tests were performed in two steps after confirmation of the reliability and validity of the measurement scales. A “PLS Algorithm” was run first to generate factor scores for the latent variables. The factor scores were then used in calculating the path coefficients with consistent bootstrapping (5000 bootstrap re-samples) to avoid artificially correlated residuals resulting from the repeated use of indicators in the model [52].

Researchers should be very cautious to report and use model fit in PLS-SEM according to Hair et al. [53], as the proposed model fit criteria are in their early stage of research and are not fully understood. However, PLS-SEM does generate the standardized root mean square residual (SRMR) and the normed fit index (NFI) that have a certain threshold (SRMR < 0.08 and NFI > 0.90).

Data analyses were performed using SmartPLS 3.3.3 [54] and IBM SPSS 27.0. Bootstrapping was used to determine statistical significance of the path coefficients, including the mediation and moderation effects. All analyses were two-tailed, and a *p* ≤ 0.05 was considered statistically significant.

## Results

### Sociodemographic characteristics of respondents

The vast majority (93.4%) of respondents worked in the department of general practice. Nearly 80% were in the age range of 30–49 years and 70% were female. Only 4% did not have a bachelor degree. About 36.6% had 15 or more years of working experience. Over two-thirds of respondents had a mid-career professional title. Only 14.8% had been involved in MCI detection and management in the past. Less than 30% reported having received MCI training, but only 4.2% were awarded qualification for MCI screening.

### Perceived extrinsic barriers

The scales measuring perceived extrinsic barriers showed satisfactory reliability, with Cronbach’s alpha, rho-A, and composite reliability coefficients all exceeding the threshold of 0.7 (Additional file 1). All of the measurement items were retained. Although the loading of one item fell below 0.7, removal of the item would not increase the composite reliability of its respective domain. The convergent and discriminatory validity of the measurement scales were confirmed by the AVE (Additional file 1) and the HTMT (Additional file 2) criteria, respectively. Overall, the data fit well into the tested SEM: SRMR = 0.066, NFI = 0.933.

On average, the study participants reported a barrier score (Mean ± Standard Deviation) of 65.23 ± 13.98, 58.34 ± 16.95, and 60.37 ± 16.99 for patient engagement, working environment, and system context, respectively. More respondents appear to agree with barriers to patient engagement. In terms of patient-engagement barriers, stigma was the most frequently (68.9%) reported, followed by a lack of confidence in GPs (68.7%). Time constraints (53.5%), a lack of effective tools (49.1%), and financial incentives (48.5%) were the most frequently reported barriers in the working environment. More than half of the respondents considered the absence of MCI management in the essential public health services package and the primary care payment scheme as a major system barrier (Table 1).

**Table 1:** Perceived extrinsic barriers reported by study participants

Perceived extrinsic barriers	Number (percentage) of respondents				
**Patient Engagement**	**Strongly agree**	**Agree**	**Unsure**	**Disagree**	**Strongly disagree**
1. Most people would take MCI as normal ageing.	4.4%	55.1%	34.5%	5.0%	1.0%
2. Most people don’t believe there exist effective methods to treat MCI.	4.2%	46.4%	40.7%	7.5%	1.1%
3. Most people believe the diagnosis of MCI would lead to stigma.	10.9%	58.0%	27.6%	2.5%	1.0%
4. Patients prefer to go to a specialist hospital or tertiary hospital if they have cognitive disorder.	7.2%	53.6%	34.6%	3.8%	0.7%
5. Most people don’t have confidence in GPs to detect and manage MCI.	8.8%	59.9%	27.7%	2.9%	0.8%
6. Only when cognition was seriously affected would people go to see a doctor.	6.6%	51.6%	38.0%	2.9%	0.9%
7. People would consider MCI screening tests are too lengthy.	8.5%	53.7%	34.0%	2.9%	0.9%
8. Most people would not detect or manage MCI with money out of pocket.	4.7%	43.1%	44.3%	7.1%	0.8%
9. Most families would not support MCI detection and management.	4.9%	49.6%	41.5%	3.2%	0.7%
10. Most families feel helpless to urge suspected patients to take MCI detection and management.	4.9%	49.8%	39.7%	4.9%	0.8%
11. The less valued by the family, the less likely people would participate in MCI detection and management.	5.2%	55.0%	35.5%	3.4%	0.9%
12. People’s negative attitudes towards life would discourage them to engage in MCI detection and management.	6.8%	56.5%	32.4%	3.6%	0.7%
13. People would not pay much attention to MCI when suffering from too many chronic diseases.	5.8%	59.9%	29.4%	3.9%	1.0%
14. Good relationship with GPs will help patients engage in MCI detection and management.	5.8%	59.9%	29.4%	3.9%	1.0%
**Working Environment**	**Strongly agree**	**Agree**	**Unsure**	**Disagree**	**Strongly disagree**
1. There exist no evidence-based guidelines designed to facilitate MCI detection and management in our community health centre.	5.3%	36.6%	45.1%	10.9%	2.1%
2. There exist no economic reimbursement in our community health centre to encourage MCI detection and management.	7.2%	41.3%	42.2%	7.3%	2.0%
3. I don’t have enough disposable time to detect and manage MCI.	8.7%	44.8%	37.1%	7.9%	1.5%
4. The institutional routine policies in our community health centre did not include the practice of detecting and managing MCI.	6.5%	41.2%	41.9%	8.6%	1.8%
5. There exists no referral pathway to encourage MCI detection and management in our community health centre.	5.6%	32.5%	42.9%	16.3%	2.7%
6. There exists no supportive team in our community health centre to help GPs to detect and manage MCI.	5.8%	35.6%	44.5%	12.2%	1.9%
7. Our community health center provides no specific training on MCI detection and management.	5.2%	33.4%	43.5%	16.1%	1.8%
8. The clinic electronic system health centre is lack function for improving MCI detection and management.	6.0%	40.4%	41.8%	9.9%	1.9%
9. There is a lack of essential tools for detecting and managing MCI in our community health centre.	6.8%	42.3%	41.3%	8.1%	1.5%
10. There are not enough facilities and space in our community health centre to detect or manage MCI in our community.	7.0%	41.5%	41.3%	8.4%	1.8%
11. There is a serious lack of human resources in our community health centre.	6.4%	28.7%	43.5%	17.7%	3.7%
12. Managers in our community health centre put less emphasis on MCI detection and management.	4.6%	23.8%	52.2%	16.3%	3.1%
**System Context**	**Strongly agree**	**Agree**	**Unsure**	**Disagree**	**Strongly disagree**
1. The government put less investment on MCI detection and management.	7.6%	36.7%	50.0%	3.5%	2.2%
2. MCI detection and management have not been accepted by the whole society.	4.7%	29.4%	45.5%	16.4%	4.0%
3. The public media focus less on the topic of MCI.	5.7%	36.5%	44.4%	11.2%	2.2%
4. There exist less publication of MCI detection and management from the public media.	6.1%	44.4%	41.1%	6.4%	2.0%
5. The primary health care system does not cover payments from MCI detection and management.	10.4%	43.3%	39.9%	4.5%	1.9%
6. The basic package of public health provides no definite stipulation on MCI detection and management.	8.1%	42.9%	41.6%	5.1%	2.3%
7. The governments’ legislative regulations did not clarify the specific roles of different professions to detect and manage MCI.	8.2%	40.5%	44.6%	4.6%	2.1%

### Correlations between KAP scores and perceived extrinsic barriers

The three domains of perceived barriers were positively correlated with each other (*p* < 0.001). Perceived barriers in patient engagement were negatively associated with MCI-related knowledge, attitudes, and intended practice of GPs (*p* < 0.001). Perceived barriers in the system context were negatively associated with MCI-related knowledge and attitudes (*p* < 0.001), compared with a marginal positive correlation between perceived barriers in the working environment and intended practice(*p* = 0.039) (Table 2).

**Table 2:** Correlation matrix of the relationship among the latent variables

	1	2	3	4	5
1. Perceived system context	1				
2. Perceived working environment	0.620^**^	1			
3. Perceived Patient engagement	0.467^**^	0.450^**^	1		
4. Intended practice	−0.002	0.058^*^	−0.116^**^	1	
5. Attitudes	−0.104^**^	−0.006	−0.312^**^	0.211^**^	1
6. knowledge	−0.075^**^	−0.008	−0.180^**^	0.212^**^	0.248^**^

^*^p = 0.039

^**^p < 0.001

### Structural equation model

Knowledge was associated with the intended practice, with the direct effect contributing to 68.2% of the total effect. The indirect effects of knowledge on intended practice via attitudes (84.6%) and perceived extrinsic barriers (− 15.4%) were both statistically significant (Additional file 3).

**Fig. 2:**
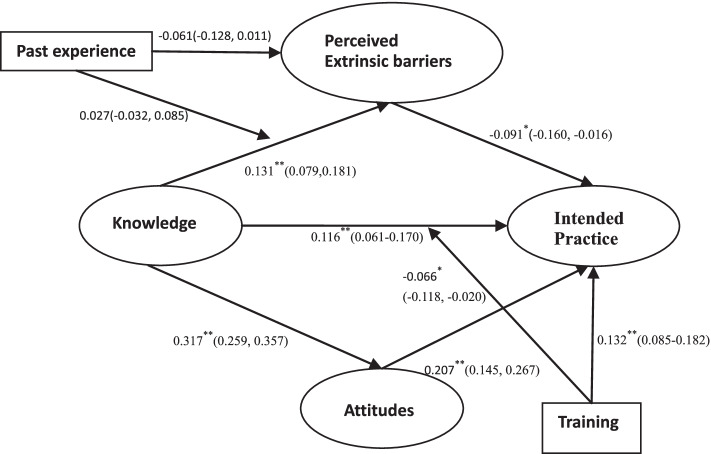
Structure equation model of perceived extrinsic barriers on KAP of MCI. Path coefficient (95% confidence intervals) are presented (*p < 0.05, ** *p <* 0.01)

While attitudes mediated the effect of knowledge on intended practice in a positive manner, the mediating effect of perceived extrinsic barriers was negative (Fig. 2). High MCI knowledge was associated with higher levels of perceived extrinsic barriers (β = 0.131, *p* < 0.001); whereas, higher perceived extrinsic barriers led to the lower intention of adherence to practice guidelines (β = − 0.091, *p* = 0.012) (Additional file 4).

**Fig. 3:**
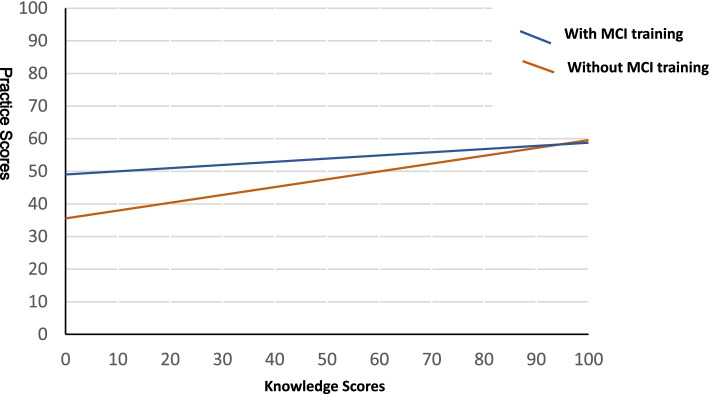
Moderation effect of training on the association between knowledge and intended practice

The training was associated with higher levels of practice compliance (β = 0.132, *p* < 0.001). It also moderated the association between knowledge and intended practice (β = − 0.066, *p* = 0.017): the effect of training was less powerful when GPs had a higher level of knowledge (Fig. 3).

Past experience had no significant relationship with perceived extrinsic barriers (β = − 0.061, *p* = 0.084), nor did it moderate the association between knowledge and perceived extrinsic barriers (β = 0.027, *p* = 0.400).

## Discussion

This study assessed the perceived extrinsic barriers and their mediating effect on the association between knowledge and intended practice of GPs in community MCI detection and management. Our study revealed that social stigma and a lack of confidence in GPs are major barriers to patient engagement as perceived by the GP respondents, while resource constraints and a lack of policy, financial and policy support are major working environment and system barriers. The perceived extrinsic barriers have a negative mediating effect on the association between knowledge and intended practice, hence, hypothesis one is supported. Training has a positive moderating effect on the association between knowledge and intended practice, and the effect is less powerful when GPs have a higher level of knowledge, hence, hypothesis two is supported. However, past experience did not show a significant effect on perceived extrinsic barriers, hence, hypothesis three is not supported.

This study indicated that both intrinsic drivers (such as knowledge and attitudes of physicians) and extrinsic drivers (patient engagement, working environment, and system context) have shaped the intended practice of primary care physicians in detecting and managing MCI. These results are consistent with the findings reported in a recent systematic review [23], which categorised barriers to dementia care into patient, provider, and system-related. The influence of system context on health practice has been widely acknowledged in health policy documents [55, 56]. The organisational factors have been identified as influencing the motivation of healthcare providers from both the perspectives of financial and non-financial incentives [27]. The average barrier score for patient engagement was found to be 65.23 out of a maximum of 100 in this study, which is the highest among the three barrier domains. A cross-sectional survey of 703 GPs in the Netherlands indicated that the most perceived barriers to implementing clinical guidelines came from external factors, especially patient preferences, needs, and abilities [57]. However, findings on improving physician guideline adherence behaviour may not be generalisable, since barriers in one setting may not be present in another.

Our SEM results showed that perceived extrinsic barriers had a negative mediating impact (β = − 0.012, *p* = 0.025) on the association between knowledge and intended practice, accounting for 15.4% of all indirect effects. A partial mediating effect was confirmed, which suggests that the K-A-P pathway remains to be a major pathway for translating knowledge into practice, and perceived extrinsic barriers have a weak but non-negligible effect on the intended practice of GPs in MCI detection and management. Therefore, there is no doubt that the intention of GPs to detect and manage MCI can be compromised when they perceive high levels of extrinsic barriers. Accordingly, it could be understandable why GPs rarely detected MCI in practice [10] even though the majority of primary care physicians acknowledged the value of assessing cognitive impairment in primary care [11].

The association between higher knowledge and higher perceived extrinsic barriers is concerning. The GPs with a high level of MCI-related knowledge are more likely to notice potential extrinsic barriers in their practice compared with those with a low level of knowledge. Those knowledgeable physicians may perceive more challenges when the process of implementing approaches impacts their routines and workflow and requires them to work in new ways. Similarly, a systematic review [58] found that workload and time constraints are dominant barriers to implementing evidence-based dementia care.

The moderation analyses showed that training can potentially improve compliance to practice guidelines. However, the effect of training is less powerful when GPs have already had a higher level of knowledge. It is important to note that knowledge is often acquired through training, but high knowledge is also associated with high perceived barriers. A systematic review of six studies [59] concludes that education alone would not increase adherence of primary care to dementia care guidelines. However, a targeted physician practice-based educational intervention along with community services support is more effective for improving the dementia care competency of clinicians according to a cohort study [28].

In this study, we did not find a significant moderation effect of past experience on the association between knowledge and perceived extrinsic barriers. It may be, at least partly, due to the fact that only 14.8% of the GP respondents reported experience with MCI detection and management. Community-based MCI management is still in its initial development stage in China. Some GPs may have obtained the experience through research or experimental studies. However, implementation or incorporation of the services into routine practice is a different matter. The additional resources available to a research project are likely to disappear. The patients receiving services may become more diversified. The widespread participation of GPs in the new initiative would require some additional incentives. Unfortunately, those who are prepared to practice are more likely to be aware of the barriers in working environment according to the findings of this study. This result is consistent with the results of a qualitative study that explored a “disconnect” between perceptions of GPs and other providers regarding the need for implementation of a chronic disease prevention program in primary care settings. GPs are likely to be more concerned about the lack of a supportive environment than their colleagues [60].

The findings of this study have some implications for policy and management as well as educational activities. Extrinsic barriers in relation to patient engagement, working environment, and system context should be addressed to provide support to GPs in community detection and management of MCI. According to Herzberg’s motivation theory [61], those extrinsic barriers are deemed as hygiene factors that can result in staff dissatisfaction if not addressed properly, even though they do not in themselves motivate employees. They may even deter the efforts of some intrinsically motivated GPs. Training remains critical given that the overall knowledge level of GPs in MCI detection and management is low. Training is particularly powerful when knowledge is low. Meanwhile, however, GPs need to be equipped with skills to adequately cope with the challenging environment. This should include, but not be limited to, more proactive engagement in patient and public education campaigns and advocacy for increasing policy and management support for community detection and management of MCI [62]. A systems approach is needed to reduce the barriers, which includes but is not limited to the alignment of policy goals, adequate funding arrangements, coordination between different levels of services, management support, and public education and community mobilisation.

### Strengths and limitations

The SEM-PLS method was adopted to explore the complex exploratory structural equation model with both formative and reflective measures. This study tested the mediating effect of perceived extrinsic barriers and the moderation effect of training on the association between MCI knowledge and intended practice, and the moderation effect of past experience on the association between MCI knowledge and extrinsic barriers in a large sample of GPs. The findings have both policy/management and training implications for developing the programs in relation to community detection and management of MCI in response to the challenges of an ageing society.

Like any other study, this study also has some limitations. Firstly, although this survey included large sample size, it did not represent the entire GP workforce. Attempts to generalise the findings of this study should be undertaken cautiously. Secondly, the concept of perceived barriers measured in this study is not equivalent to the objective existence of extrinsic barriers. Data were collected through self-reporting, which is subject to reporting bias. However, perceived extrinsic barriers bear a more direct connection with practice intentions [13]. Finally, we took intended practice to be an outcome measurement, since there exist limited actual MCI detection and management activities including those originating from research and experimental projects. However, intended practice has been regarded as the most immediate predictor of actual practice, even though there exists an intention-behaviour gap [63]. A meta-analysis of 10 meta-analyses concluded that intention accounts for almost one-third of the variance in behaviour [64].

## Conclusions

Perceived extrinsic barriers jeopardise the translation of MCI knowledge into intended actions that comply with practice guidelines in GPs in Shanghai. Although intrinsic drivers account for the majority of indirect effects between knowledge and intended practice, perceived extrinsic barriers negatively mediate the association between knowledge and practice. Training can improve practice; however, its effect is more powerful when the knowledge level is low. Training alone is not enough as increased knowledge can be associated with higher perceived extrinsic barriers. Extrinsic barriers need to be addressed to support the efforts of GPs in community detection and management of MCI. Training should be prioritised for those with lower MCI knowledge, and enhance the skills of GPs to adequately cope with the challenging environment.

## Supplementary Information

**Additional file 1: Table S1.** Reliability and validity of Reflective Measurement Models.

**Additional file 2: Table S2.** Discriminant validity (HTMT) of the scales measuring perceived extrinsic barriers.

**Additional file 3: Table S3.** Indirect effects of knowledge on intended practice.

**Additional file 4.** Path coefficients and hypothesis testing.

## Data Availability

The datasets generated and analysed during the current study are not publicly available due to policies from the ethics committee, but are available from the corresponding author CL on reasonable request.

## Abbreviations

GPs: General practitioners
MCI: Mild cognitive impairment
KAP: Knowledge, attitudes and practice
CHCs: Community health centres
CCM: The Chronic Care Model
