# Supplementary material for: Perceived extrinsic barriers hinder community detection and management of mild cognitive impairment: a cross-sectional study of general practitioners in Shanghai, China
Source: BMC Geriatr. 2022 Jun 9;22:497. doi: 10.1186/s12877-022-03175-4 (PMC9185915; doi:10.1186/s12877-022-03175-4)
Supplement: Supplementary file 1 — Additional file 1: Table S1. Reliability and validity of Reflective Measurement Models. [file 12877_2022_3175_MOESM1_ESM.docx]

**Table S1. Reliability and validity of Reflective Measurement Models**

| **Latent Variables** | **Indicators** | **Loadings** | **Cronbach’s alpha** | **rho_A** | **Composite Reliability** | **Average Variance Explained** |
| --- | --- | --- | --- | --- | --- | --- |
| **Test criteria** |  | **>0.7** | **> 0.70** | **> 0.70** | **> 0.70** | **> 0.50** |
| **Patient engagement** | R-1 | 0.756 | 0.955 | 0.957 | 0.960 | 0.634 |
|  | R-2 | 0.781 |  |  |  |  |
|  | R-3 | 0.713 |  |  |  |  |
|  | R-4 | 0.807 |  |  |  |  |
|  | R-5 | 0.786 |  |  |  |  |
|  | R-6 | 0.849 |  |  |  |  |
|  | R-7 | 0.812 |  |  |  |  |
|  | R-8 | 0.827 |  |  |  |  |
|  | R-9 | 0.743 |  |  |  |  |
|  | R-10 | 0.826 |  |  |  |  |
|  | R-11 | 0.793 |  |  |  |  |
|  | R-12 | 0.842 |  |  |  |  |
|  | R-13 | 0.845 |  |  |  |  |
|  | R-14 | 0.756 |  |  |  |  |
| **System context** | S-1 | 0.833 | 0.930 | 0.939 | 0.944 | 0.710 |
|  | S-2 | 0.833 |  |  |  |  |
|  | S-3 | 0.853 |  |  |  |  |
|  | S-4 | 0.863 |  |  |  |  |
|  | S-5 | 0.884 |  |  |  |  |
|  | S-6 | 0.900 |  |  |  |  |
|  | S-7 | 0.901 |  |  |  |  |
| **Working environment** | O-1 | 0.842 | 0.954 | 0.958 | 0.961 | 0.672 |
|  | O-2 | 0.851 |  |  |  |  |
|  | O-3 | 0.769 |  |  |  |  |
|  | O-4 | 0.882 |  |  |  |  |
|  | O-5 | 0.768 |  |  |  |  |
|  | O-6 | 0.862 |  |  |  |  |
|  | O-7 | 0.812 |  |  |  |  |
|  | O-8 | 0.866 |  |  |  |  |
|  | O-9 | 0.876 |  |  |  |  |
|  | O-10 | 0.878 |  |  |  |  |
|  | O-11 | 0.596 |  |  |  |  |
|  | O-12 | 0.788 |  |  |  |  |
