# Supplementary material for: Perceived extrinsic barriers hinder community detection and management of mild cognitive impairment: a cross-sectional study of general practitioners in Shanghai, China
Source: BMC Geriatr. 2022 Jun 9;22:497. doi: 10.1186/s12877-022-03175-4 (PMC9185915; doi:10.1186/s12877-022-03175-4)
Supplement: Supplementary file 2 — Additional file 2: Table S2. Discriminant validity (HTMT) of the scales measuring perceived extrinsic barriers. [file 12877_2022_3175_MOESM2_ESM.docx]

**Table S2. Discriminant validity (HTMT) of the scales measuring perceived extrinsic barriers**

|  | Patient engagement | System context | Perceived extrinsic barriers |
| --- | --- | --- | --- |
| System context | 0.497 |  |  |
| Perceived extrinsic barriers | 0.867 | 0.824 |  |
| Working environment | 0.473 | 0.659 | 0.875 |
