# Supplementary material for: Perceived extrinsic barriers hinder community detection and management of mild cognitive impairment: a cross-sectional study of general practitioners in Shanghai, China
Source: BMC Geriatr. 2022 Jun 9;22:497. doi: 10.1186/s12877-022-03175-4 (PMC9185915; doi:10.1186/s12877-022-03175-4)
Supplement: Supplementary file 3 — Additional file 3: Table S3. Indirect effects of knowledge on intended practice. [file 12877_2022_3175_MOESM3_ESM.docx]

**Table S3. Indirect effects of knowledge on intended practice**

| **Path** | **Indirect effect** | **95% Confidence Interval** | | ***p*** |
| --- | --- | --- | --- | --- |
|  |  | **Lower** | **Upper** |  |
| Knowledge-> Attitudes->Intended behaviour | 0.066 | 0.046 | 0.087 | <0.001 |
| Knowledge->Perceived Extrinsic barriers->Intended behaviour | -0.012 | -0.025 | -0.003 | 0.025 |
