# Supplementary material for: Perceived extrinsic barriers hinder community detection and management of mild cognitive impairment: a cross-sectional study of general practitioners in Shanghai, China
Source: BMC Geriatr. 2022 Jun 9;22:497. doi: 10.1186/s12877-022-03175-4 (PMC9185915; doi:10.1186/s12877-022-03175-4)
Supplement: Supplementary file 4 — Additional file 4. Path coefficients and hypothesis testing. [file 12877_2022_3175_MOESM4_ESM.docx]

**Additional file 4. Path coefficients and hypothesis testing**

| **Path** | **Path coefficient** | ***t*** | ***p*** | **95%CI LL** | **95%CI UL** |
| --- | --- | --- | --- | --- | --- |
| Knowledge->Intended behaviour | 0.116 | 3.865 | <0.001 | 0.061 | 0.17 |
| Knowledge-> Attitudes | 0.317 | 12.631 | <0.001 | 0.259 | 0.357 |
| Attitudes->Intended behaviour | 0.207 | 6.851 | <0.001 | 0.145 | 0.267 |
| Knowledge-> Perceived extrinsic barriers | 0.131 | 5.000 | <0.001 | 0.079 | 0.181 |
| Perceived extrinsic barriers->Intended behaviour | -0.091 | 2.525 | 0.018 | -0.160 | -0.016 |
| Past experience-> Perceived extrinsic barriers | -0.061 | 1.73 | 0.084 | -0.128 | 0.011 |
| Moderating effect of past experience-> Perceived extrinsic barriers | 0.027 | 0.851 | 0.400 | -0.032 | 0.085 |
| Training ->Intended behaviour | 0.132 | 5.132 | <0.001 | 0.085 | 0.182 |
| Moderating effect of training->Intended behaviour | -0.066 | 2.575 | 0.014 | -0.118 | -0.020 |
